# Clinical study of wearable low-intensity pulsed ultrasound treatment in the improvement of executive function in obesity (the SLITE trial): protocol for a single -centre, double-blind, randomized controlled trial

**DOI:** 10.3389/fnhum.2025.1698351

**Published:** 2025-10-24

**Authors:** Xiaoxian Xiong, Chuanyu Zhong, Tingyu Zhang, Xue Xie, Junnian Hao, Yuanyi Zheng, Fengjing Liu

**Affiliations:** ^1^Department of Ultrasound in Medicine, Shanghai Sixth People’s Hospital Affiliated to Shanghai Jiao Tong University School of Medicine, Shanghai, China; ^2^Shanghai Center for Brain Science and Brain-Inspired Technology, Shanghai, China; ^3^Department of Endocrinology and Metabolism, Shanghai Sixth People’s Hospital Affiliated to Shanghai Jiao Tong University School of Medicine, Shanghai, China

**Keywords:** obesity, low-intensity pulsed ultrasound, executive function, neuromodulation, randomized controlled trial

## Abstract

**Introduction:**

Obesity is a global health crisis associated with significant physical and cognitive impairments, particularly in executive function. Impaired executive function exacerbates unhealthy eating behaviors and hinders effective weight management. Non-invasive neuromodulation techniques like transcranial direct current stimulation and transcranial magnetic stimulation have demonstrated potential in improving executive function and assisting in weight reduction but face limitations in accessibility and efficacy. Low-intensity pulsed ultrasound (LIPUS) represents an emerging neuromodulation technique distinguished by its non-invasive application and superior spatial resolution, facilitating targeted modulation of cortical activity and neural excitability with high precision. This study aims to explore the efficacy of LIPUS on executive function in obesity.

**Methods and analysis:**

This study adopts a prospective, single-center, double-blind, randomized controlled design. A total of 44 participants diagnosed with obesity (BMI ≥ 28 kg/m^2^) will be enrolled and equally assigned to the LIPUS and sham groups. The LIPUS group will receive pulsed therapeutic ultrasound for 4 weeks, while the sham group will receive placebo treatment using a device delivering no therapeutic ultrasound. Both groups will undergo assessments at baseline and after 4 weeks, with the primary outcome being the change in reaction time on the food Go/No-go task. Secondary outcomes include changes in body weight, BMI, food cravings (measured by the Food Craving Questionnaire-Trait and State versions), food-eating behavior (measured by the Visual Analog Scale and the Dutch Eating Behavior Questionnaire), and cognitive performance as assessed by the Stroop test. Additional neuroimaging assessments will include functional near-infrared spectroscopy (fNIRS), functional magnetic resonance imaging (fMRI), and electroencephalography (EEG).

**Discussion:**

This trial will be the first to evaluate LIPUS for enhancing executive function in individuals with obesity. By combining wearable ultrasound technology with neuroimaging assessments, the study is expected to provide novel evidence for the neuromodulatory role of LIPUS. The results may support the development of innovative, non-invasive strategies for obesity management and inform future clinical applications.

## 1 Introduction

Obesity, characterized by an excessive accumulation of fat tissue that negatively impacts health, has been escalating into a global health crisis, posing significant challenges to public health systems (Lin and Li, 2021). According to the World Health Organization, obesity rates more than doubled between 1990 and 2022 (Obesity and Collaborators, 2024), reflecting its widespread impact across various age groups and socioeconomic backgrounds. Beyond its well-documented physical consequences, such as cardiovascular diseases and type 2 diabetes (Piché et al., 2020; Caballero, 2019; Seravalle and Grassi, 2017), obesity is increasingly recognized for its adverse effects on cognitive functions, with growing evidence linking excess body fat to impairments in memory, attention, and executive function (Mattson, 2019; Lowe et al., 2019). Conversely, the decline in executive function, which governs planning, decision-making, and self-regulation, can make it more difficult for individuals to adhere to healthy behaviors, such as managing diet and exercise, contributing to a vicious cycle of weight gain and reduced quality of life (Doebel, 2020; Yang et al., 2018). Thus, the decline of executive function in obesity, as a crucial cognitive domain, calls for immediate focus and intervention from both public health organizations and healthcare professionals.

Executive function plays a crucial role in translating healthy eating principles into daily behaviors and sustaining the behavioral changes necessary to overcome obesity (Yang et al., 2018). Evidence suggests that deficiencies in executive function, especially with cognitive flexibility and inhibitory control, are closely associated with poor weight management outcomes (Foldi et al., 2021). Despite the growing recognition of its importance, effective interventions specifically targeting executive function deficits in individuals with obesity remain limited. Evidence highlights that the dorsolateral prefrontal cortex (DLPFC), a neural hub responsible for executive function regulation and control, typically shows reduced activation after meals in obese individuals compared to lean individuals (Le et al., 2007). Moreover, evidence indicates that enhancing DLPFC activity can improve decision-making, impulse control, and dietary compliance among individuals with obesity (Gluck et al., 2017; Kringelbach et al., 2004). Therefore, developing interventions to modulate DLPFC activity holds promise for addressing executive function-related impairments in obese population, which may help accelerate the prevention and treatment of obesity.

In recent years, non-invasive brain stimulation techniques, such as transcranial direct current stimulation (tDCS) and transcranial magnetic stimulation (TMS), have shown potential in improving cognitive function and eating behavior in obesity (Kekic et al., 2014). For instance, home-based tDCS targeting the dorsolateral prefrontal cortex (DLPFC) was shown to significantly reduce uncontrolled and emotional eating in patients with fibromyalgia (Jornada et al., 2024), while Anderson et al. (2023) reported that tDCS over the DLPFC altered the perceived pleasantness of food stimuli (Anderson et al., 2023). A recent randomized controlled trial further explores tDCS modulation of brain reactivity to food cues in obesity (Ghobadi-Azbari et al., 2022). Similarly, repetitive TMS (rTMS) applied to the left DLPFC improved food craving control and eating behaviors in obese participants and contributed to modest weight reduction (Kim et al., 2018). Despite these encouraging findings, both modalities have notable limitations. The efficacy of tDCS remains inconsistent, largely due to inter-individual variability in neural responsiveness, which hampers its predictability and generalizability (Vergallito et al., 2022; Evans et al., 2020). Similarly, TMS is constrained by limited portability and intrinsic challenges in achieving fine spatial precision and depth selectivity, which may reduce accessibility and hinder accurate targeting of specific cortical regions (Bhattacharya et al., 2022).

Low-intensity pulsed ultrasound (LIPUS) is an emerging non-invasive therapeutic modality that applies ultrasound waves with low intensity and pulsed frequency to stimulate tissues at a cellular level. Traditionally used in musculoskeletal rehabilitation for its ability to promote tissue healing and reduce inflammation (Qin et al., 2023), LIPUS has recently attracted attention for its potential in modulating brain activity and improving cognitive function (Zeng et al., 2022). By fine-tuning parameters such as intensity, frequency, pulse width, and duration, LIPUS enables bidirectional modulation of targeted brain areas (Dallapiazza et al., 2018), including the DLPFC. Compared to traditional neuromodulation techniques, LIPUS offers deeper tissue penetration and localized stimulation without the need for invasive procedures (Tufail et al., 2011). Moreover, LIPUS operates intermittently, minimizing thermal effects and allowing precise stimulation without damaging surrounding healthy tissue (Fomenko et al., 2018). Research has demonstrated the potential of LIPUS to improve cognitive function in diverse groups, including individuals with Alzheimer’s disease (Shimokawa et al., 2022), as well as those with traumatic brain injury (Wang W. et al., 2024; Su et al., 2017). However, no studies have yet investigated the application of transcranial ultrasound therapy for improving executive function in individuals with obesity.

Therefore, the purpose of this study is to explore the efficacy of LIPUS as a potential non-invasive intervention to improve executive function in obesity, utilizing a self-developed wearable LIPUS device specifically designed for this purpose. We hypothesize that 4 weeks of daily LIPUS stimulation targeting the DLPFC will enhance executive function, as reflected by shorter reaction time in the Food Go/No-go task in the LIPUS group, whereas no significant changes are expected in the sham group. Furthermore, these improvements in executive function are expected to facilitate reductions in body weight and BMI.

## 2 Methods

### 2.1 Study design

**FIGURE 1:**
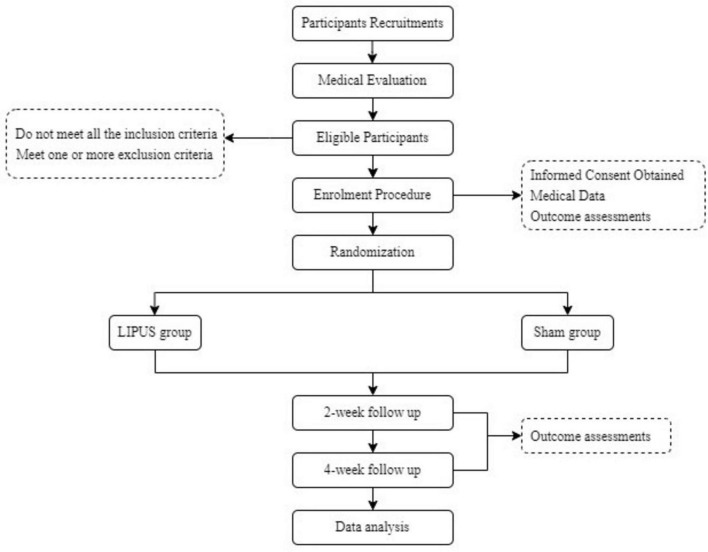
Participant flow chart.

This trial is a prospective, single-center, double-blind randomized controlled trial (RCT) to be conducted at the Shanghai Jiao Tong University of Medicine Affiliated Sixth People’s Hospital. Eligible participants will be identified based on a diagnosis of obesity (BMI ≥ 28 kg/m^2^) according to Chinese criteria (Zeng et al., 2021). Participants will be evenly randomized in a 1:1 ratio into either the LIPUS group or the sham group. The trial design will be conducted following the Standard Protocol Items: Recommendations for Interventional Trials guidelines (SPIRIT; Chan et al., 2013), and the detailed study workflow is depicted in Figure 1.

### 2.2 Participant and public involvement

Participants and the general public were not directly involved in framing the research question, selecting outcome measures, designing the study, recruiting participants, or overseeing its implementation. However, upon the completion of the trial, results will be shared with participants and the public through accessible formats.

### 2.3 Participant recruitment

A total of 44 participants diagnosed with obesity (BMI ≥ 28 kg/m^2^) will be enrolled and equally assigned to the LIPUS and sham groups. Recruitment is scheduled to commence on 10 December 2024 and continue over a span of 15 months or until the target sample size is achieved. Recruitment activities will be conducted through the endocrinology outpatient clinics at Shanghai Jiao Tong University of Medicine Affiliated Sixth People’s Hospital, Shanghai, China. Prospective participants and their guardians will be invited to an in-person meeting, during which the study’s objectives, procedures, potential benefits, and any associated risks or discomforts will be explained in detail. Participants will be required to provide written consent before being formally enrolled in the study.

### 2.4 Inclusion/exclusion criteria

#### 2.4.1 Inclusion criteria

- BMI ≥ 28 kg/m^2^ and <35 kg/m^2^;
- Aged 18–60 years, inclusive;
- The subject or the subject’s family agrees and signs an informed consent form, being able to cooperate with various examinations during the clinical research period.

#### 2.4.2 Exclusion criteria

- History of head trauma or epilepsy, or a family history of epileptic seizures;
- Presence of implanted metal devices, pacemakers, claustrophobia, or any other contraindications to functional magnetic resonance imaging (fMRI);
- Existence of diabetes mellitus or unstable cardiovascular diseases;
- Greater than 5% change in body weight within the past 3 months;
- Receipt of weight loss treatment within the past 6 months;
- Diagnosis of psychiatric disorders or current use of psychotropic medications;
- Abnormal findings on brain magnetic resonance imaging (MRI);
- Severe hepatic or renal dysfunction;
- Inability to communicate with researchers or staff, or participation in other clinical studies simultaneously.

### 2.5 Medical evaluation and enrollment procedure

Participants will undergo a screening process to assess their eligibility for the trial. This will include a thorough health history review and a physical examination to ensure they meet the inclusion criteria and do not have any conditions that would exclude them from participation.

Following the medical evaluation, baseline data will be collected from all eligible participants, including demographic information (age, gender, and occupation), lifestyle habits (smoking and alcohol consumption), body weight, body mass index (BMI), and medical history. Also, participants will be assessed using the food Go/No-go task and the Stroop task, as well as rating scales including the Food Cravings Questionnaire-Trait (FCQ-T) and Food Cravings Questionnaire-State (FCQ-S), visual analog scale (VAS), Dutch Eating Behavior Questionnaire (DEBQ). Other medical examinations will be performed, including functional near-infrared spectroscopy (fNIRS), electroencephalogram (EEG), and functional magnetic resonance imaging (fMRI).

### 2.6 Randomization and blinding

Randomization will be carried out by an independent statistician, who is not involved in the implementation or analysis of this trial, using a random number table generated by the Microsoft Excel. Participants will be randomly assigned to either the LIPUS group or the sham group in equal proportions (1:1) using a block randomization method. To ensure allocation concealment, group assignments will be sealed in opaque, sequentially numbered envelopes.

Blinding will be maintained for both participants and study staff involved in data collection, outcome assessment and statistical analysis. All LIPUS treatment devices are equipped with a vibration mode, rendering no discernible difference between the LIPUS and sham group during the trial. Blinding integrity will be verified periodically during the trial to ensure adherence.

### 2.7 Intervention

**FIGURE 2:**
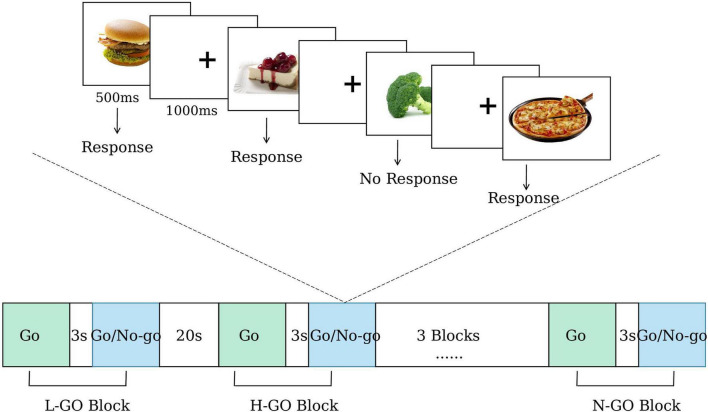
External view of the low-intensity pulsed ultrasound (LIPUS) device. Food images reproduced from Blechert et al. (2019).

Participants will perform LIPUS therapy independently at home over a 4-week period, utilizing a self-developed wearable device (Figure 2). The LIPUS group will receive pulsed therapeutic ultrasound, targeting the bilateral DLPFC projections on the scalp. The LIPUS device operates at a frequency of 600 kHz with an effective intensity of 1.0 W/cm^2^ and a 50% duty cycle (10 ms on, 10 ms off). Two transducers work alternately (each active for 1 s) during a 20-min treatment session, maintaining the treatment-site temperature below 41 °C to ensure safe and stable acoustic stimulation (Hayner and Hynynen, 2001; Zhang et al., 2021; U.S. Food and Drug Administration, 2019; Fomenko et al., 2020; Legon et al., 2020; Pellow et al., 2024). Each session will last 20 min, performed daily over 4 weeks. Treatment settings will remain unchanged throughout the trial unless the participant’s condition worsens or adverse effects are observed. The sham group will use a device identical in appearance and operation to the LIPUS group’s device but delivering no therapeutic ultrasound. All participants, regardless of group allocation, will receive standardized guidance on a low-calorie diet and maintenance of their habitual physical activity. This guidance is consistent with participants’ usual routines, and no additional dietary or exercise interventions will be applied. Participants will remain at home throughout the study, and will be asked to maintain daily logs of food intake and physical activity, which will be collected and reviewed regularly by the study team throughout the study period, ensuring that any observed differences in outcomes can be attributed to LIPUS stimulation.

Prior to the first session, each participant will have the positions of bilateral DLPFC projections on the scalp determined by research personnel using the international EEG 10–20 system of electrode placement (Fabregat-Sanjuan et al., 2022). The left DLPFC is typically located at position F3 and the right at position F4 (Beam et al., 2009). Once the locations are identified, the stimulation sites will be marked and a photograph will be taken. Following that, the wearable device will be positioned over the marked target areas during subsequent sessions to ensure precise ultrasound treatment.

### 2.8 Data management

As outlined in Table 1, data will be systematically collected at baseline, and at the second and fourth weeks of the treatment period. All paper-based materials, including the study protocol, case report forms and informed consent forms will be securely stored by the principal investigator in a locked box at Shanghai Jiao Tong University of Medicine Affiliated Sixth People’s Hospital. All electronic data will be securely recorded in a password-protected electronic database, accessible only to authorized team members. Participant withdrawal from the study will be permitted under specific conditions, such as voluntary withdrawal of consent or the identification of exclusion criteria post-enrollment. For all withdrawals, the reasons and dates will be meticulously documented.

**TABLE 1:** Study evaluation procedures and timeline.

Study procedure	Medical evaluation	Enrollment visit	2 weeks	4 weeks
Determine eligibility	√	√		
Obtain signed consent		√		
Other medical and demographic data		√		
Give instructions for LIPUS devices		√		
**Outcome measures**				
Food Go/No-go task		√	√	√
Body weight, BMI		√	√	√
FCQ/T, FCQ/S, VAS, DEBQ scale		√	√	√
Stroop task		√	√	√
EEG		√	√	√
fNIRS		√	√	√
fMRI		√		√

### 2.9 Outcome measures

An outline of the study outcome assessments and corresponding descriptions of their purposes is presented in Table 2.

**TABLE 2:** Outcome assessments and their respective purposes.

Outcome measures	Assessment type	Purpose description
Food Go/No-go task	Primary outcome	To assess food-related inhibitory control
Body weight and BMI	Secondary outcome	To monitor physical changes related to the intervention
FCQ-T and FCQ-S	Secondary outcome	To evaluate long-term and situational food cravings, respectively
VAS	Secondary outcome	To measure appetite and hunger perceptions
DEBQ	Secondary outcome	To assess habitual patterns of emotional, external, and restrained eating
Stroop task	Secondary outcome	To measure cognitive flexibility and selective attention
fNIRS	Secondary outcome	To measure task-related hemodynamic responses in the cortical surface
EEG	Secondary outcome	To monitor cortical activity during ultrasound neuromodulation of the DLPFC
fMRI	Secondary outcome	To evaluate changes in brain structure and connectivity

LIPUS, low-intensity pulsed ultrasound; BMI, body mass index; FCQ-T, Food Cravings Questionnaire-Trait; FCQ-S, Food Cravings Questionnaire-State; VAS, visual analog scale; DEBQ, Dutch Eating Behavior Questionnaire; EEG, electroencephalography; fNIRS, functional near-infrared spectroscopy; fMRI, functional magnetic resonance imaging.

#### 2.9.1 Primary outcome measures

The primary outcome is the difference in mean reaction time change on the food Go/No-go task from baseline to the fourth week of treatment (LIPUS group vs. sham group). The Go/No-go task is a well-recognized experimental paradigm for assessing executive functions (Raud et al., 2020; Foland-Ross et al., 2019). The food Go/No-go task is a modified version designed specifically to assess food-related inhibitory control (He et al., 2019; Wang K. et al., 2024). Key indicators for assessment include reaction time and the number of erroneous responses (Labonté and Nielsen, 2023). Shorter reaction time and fewer erroneous responses are indicative of better executive function.

In this study, each participant will complete six blocks, which are randomly assigned in equal numbers to three experimental conditions: (1) low-calorie food Go, (2) high-calorie food Go, and (3) neutral item Go. In the low-calorie food Go condition, participants are instructed to press a key in response to low-calorie food images while inhibiting responses to high-calorie food images. Conversely, in the high-calorie food Go condition, participants respond to high-calorie food images and inhibit responses to low-calorie food images. In the neutral item Go condition, participants respond to neutral non-food images and inhibit responses to all food-related images.

Each block consists of two phases: a Go-only phase (8 trials) followed by a Go/No-go phase (32 trials, Go: No-go ratio = 3:1). Each stimulus is presented for 500 ms, followed by a 1000 ms fixation cross. The interval between the Go-only and Go/No-go phases is 3 s, and the inter-block interval is 20 s. As a result, each block lasts 63 s, and the total task duration is 498 s.

**FIGURE 3:**
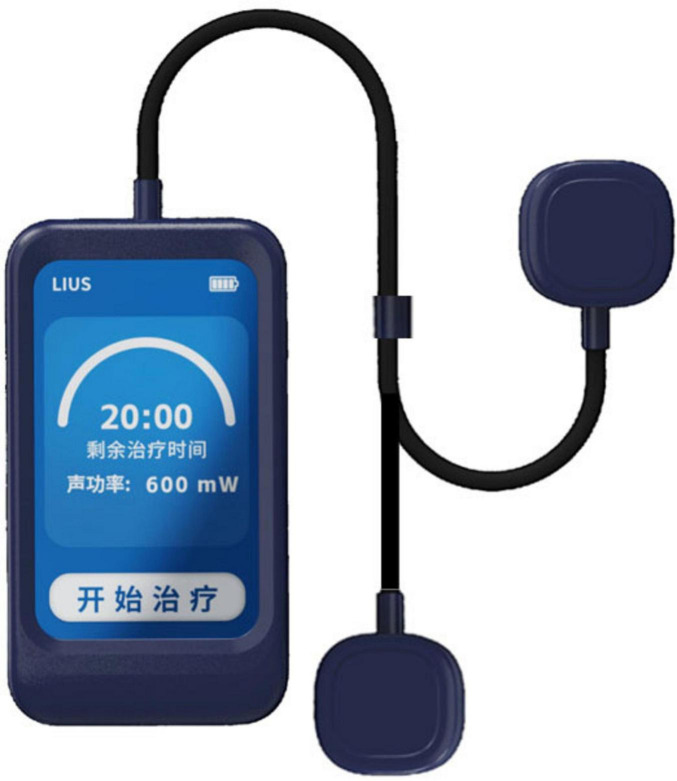
The block design of food Go/No go task.

During the task, mean reaction time for correct Go trials and commission error rates for No-go trials are recorded. In addition, fNIRS is employed to capture cortical hemodynamic responses, enabling further investigation of neural activity patterns under different task conditions. The flow of the paradigm is shown in Figure 3.

#### 2.9.2 Secondary outcome measures

- Body Weight and BMI will be used to monitor physical changes related to the intervention and will be measured at baseline, 2 weeks, and 4 weeks;
- FCQ-T and FCQ-S will be used to evaluate long-term and situational food cravings, respectively (Ulrich et al., 2016). Both will be administered at baseline, 2 weeks, and 4 weeks;
- VAS will be used to measure appetite and hunger perceptions and will be recorded at baseline, 2 weeks, and 4 weeks;
- DEBQ will be used to assess habitual patterns of emotional, external, and restrained eating and will be administered at baseline, 2 weeks, and 4 weeks.
- Stroop Task will be used to measure cognitive flexibility and selective attention by evaluating reaction time in conflicting stimulus conditions (Kalanthroff et al., 2018). Testing will occur at baseline, 2 weeks, and 4 weeks;
- fNIRS Acquisition

Functional near-infrared spectroscopy will be used to measure task-related hemodynamic responses in the cortical surface at baseline, 2 weeks, and 4 weeks (Rösch et al., 2020). Participants will wear a probe cap positioned according to the international 10–20 system, with optodes placed over the frontal and fronto-temporal regions to ensure adequate coverage of the bilateral DLPFC. Data will be collected using the ETG-4000 optical topography system (Hitachi Medical Corporation, Japan), which operates with continuous-wave near-infrared light at two wavelengths (695 and 830 nm) and a sampling rate of 10 Hz. The probe arrangement will consist of a 3 × 11 array (17 emitters and 16 detectors), providing 52 measurement channels. Raw optical density signals will be transformed into concentration changes of oxy-hemoglobin, deoxy-hemoglobin and total hemoglobin using the modified Beer–Lambert law. Channel locations will be registered to standard brain space using virtual registration procedures for subsequent group-level analysis.

Task presentation and timing will be controlled by E-Prime software (Psychology Software Tools, USA), which will send triggers to the ETG-4000 system to maintain precise synchronization between stimulus delivery and fNIRS recordings.

- EEG Acquisition

Electroencephalography will be used to monitor cortical activity during ultrasound neuromodulation of the DLPFC at baseline, 2 weeks, and 4 weeks (Schlögl et al., 2016). Resting-state signals will be recorded continuously for 5 min before stimulation (baseline) and for 5 min immediately after stimulation. A 64-channel cap configured according to the international 10–20 system will be used, sampled at 1,000 Hz with electrode impedances kept <5 kΩ.

- fMRI Acquisition

Functional magnetic resonance imaging will be used to evaluate changes in brain structure and connectivity. Scans will be performed at baseline and 4 weeks (Schlögl et al., 2016). Scanning will be performed on a 3T scanner with a 32-channel receive head coil. Each participant will first undergo high-resolution structural imaging for anatomical reference and spatial normalization. A multi-echo T1-weighted MPRAGE volume will be acquired (0.8 mm isotropic; TR = 2,500 ms; TEs = 1.81/3.60/5.39/7.18 ms; TI = 1,000 ms; flip angle = 8°; FOV = 256 mm; in-plane acceleration = 2), followed by a T2-weighted scan matched in resolution (0.8 mm isotropic; TR = 3,200 ms; TE = 564 ms; FOV = 256 mm; acceleration = 2). Auto-alignment to the AC-PC plane and vendor-supplied B0 shimming will be used prior to acquisition.

Resting-state fMRI will be collected with a multi-echo, multi-band gradient-echo EPI sequence (2.5 mm isotropic; TR = 1,670 ms; TEs = 15.6/38.2/60.8/83.4 ms; multiband factor = 4; in-plane acceleration = 2; echo spacing = 0.50 ms). To enable susceptibility-distortion correction, two runs (∼9 min, ∼300 volumes each) will be acquired with reversed phase-encoding directions (A→P and P→A) while keeping all other parameters identical. Participants will be instructed to keep their eyes open, fixate a central cross on a black background, remain awake, and minimize overt cognitive strategies. Head motion will be reduced using foam padding and a forehead strap; MR-compatible headphones/earplugs will provide acoustic attenuation.

### 2.10 Safety consideration

Participant safety will be closely monitored from the initiation of treatment (LIPUS or sham) through the 4-week follow-up period. Adverse events, the primary safety endpoint, will be documented through spontaneous participant reports, responses to specific inquiries, and observations made during general examinations conducted at biweekly study visits.

Safety will be closely monitored throughout the study by maintaining a record of adverse events, including details on the timing, severity, actions taken, relationship to treatment, and duration. No adverse effects of LIPUS have been reported in existing literature. Nonetheless, thorough documentation and consistent monitoring will be implemented to promptly address any safety concerns.

### 2.11 Sample size calculation

The sample size calculation was based on the primary outcomes. *A priori* power analysis was conducted using G*Power. Drawing from the previous trial, an F effect size of 0.25 was selected, with a conservative correlation of 0.4 between repeated measures and non-sphericity correction of 1 (Stinson et al., 2022; Kang, 2021; Wen et al., 2024). The statistical power was targeted at 80%, with a two-sided significance level (α) of 0.05. Using PASS software, the required sample size per group was determined to be n1 = n2 = 20. Considering a potential dropout rate of 10%, the adjusted total sample size includes 44 participants, evenly distributed between the two groups.

### 2.12 Analysis plan

#### 2.12.1 Statistical analysis

Data will be processed and analyzed using SPSS 26.0. Continuous data will be summarized as means and standard deviations if they follow a normal distribution; otherwise, medians and interquartile ranges will be reported. Categorical variables will be presented as frequencies and percentages. To compare continuous data between groups, independent samples *t*-tests will be used for normally distributed variables, while non-parametric tests will be applied for data that do not meet normality assumptions. For categorical data, group differences in proportions will be assessed using chi-square tests or Fisher’s exact test, depending on the data characteristics. A repeated-measures ANOVA will be conducted to evaluate outcome variables between the two groups across the three assessment time points. For the primary outcome, measured by the food Go/No-Go task, will be analyzed using an intention-to-treat approach, which includes all randomized participants. Sensitivity analyses will be conducted using per-protocol sets to examine data from participants who completed the trial as planned and adhered to the protocol, as well as full analysis sets to evaluate the robustness and generalizability of the results. Additionally, safety sets will be utilized to analyze adverse events throughout the trial.

#### 2.12.2 fNIRS data analysis

Functional near-infrared spectroscopy data will be preprocessed and analyzed using the NIRS-SPM toolbox in MATLAB. Raw optical density signals will first undergo quality assessment to identify and remove channels with excessive noise or poor signal quality, followed by motion artifact detection and correction, detrending, and band-pass filtering (0.01–0.1 Hz) to suppress low-frequency baseline drifts and high-frequency physiological noise such as heart rate and respiration. The preprocessed signals will then be converted into changes in oxy-hemoglobin and deoxy-hemoglobin concentrations using the modified Beer–Lambert law (Obrig and Villringer, 2003).

For task-related analysis, triggers sent at the onset of the Go and Go/No-Go phases within each block will be used to define events and construct an event-related design matrix. This design matrix will be convolved with a canonical hemodynamic response function, and a general linear model will be applied to estimate the amplitude of task-evoked hemodynamic responses for each phase at the individual subject level. The resulting parameter estimates will then be used for within-subject comparisons between Go and Go/No-Go phases, and for group-level statistical analyses to identify cortical regions showing significant task-related activation, with appropriate correction for multiple comparisons where necessary.

#### 2.12.3 EEG data analysis

Electroencephalography data will be processed in MATLAB/EEGLAB. After channel-wise quality control, electrodes exhibiting excessive noise or unstable impedance will be excluded. Ocular and motion artifacts will be identified and removed using independent component analysis. The data will then zero-phase band-pass filtered (0.5–45 Hz) and notch-filtered at 50 Hz to suppress line noise, before segmentation into pre- and post-stimulation epochs of equal duration.

For spectral characterization, power spectral density will be estimated with Welch’s method (Welch, 1967), and relative band power will be derived for delta (1–4 Hz), theta (4–8 Hz), alpha (8–13 Hz), beta (13–30 Hz), and gamma (30–45 Hz) ranges. Functional coupling between electrodes overlying the DLPFC and distributed cortical sites will be quantified using coherence and phase-locking value metrics. Time–frequency dynamics and spatial synchrony will be further summarized using event-related spectral perturbation and global field power, respectively.

Within-subject pre- versus post-stimulation differences will be assessed with paired statistical tests (paired *t*-test or Wilcoxon signed-rank test depending on normality), with false discovery rate control applied for multiple comparisons.

#### 2.12.4 Resting-state fMRI data analysis

Functional series will be realigned (6-dof) and distortion-corrected with the reversed phase-encode pair (TOPUP), co-registered to each participant’s T1, normalized to MNI152NLin2009cAsym, and smoothed with a 6-mm FWHM kernel. Echoes will be T2*-weighted optimally combined and denoised with ME-ICA (tedana), retaining BOLD-like components. Nuisance regression will model 6 motion parameters and their derivatives plus WM/CSF aCompCor components; high-motion volumes will be flagged as spikes when FD > 0.5 mm or DVARS exceeds the dataset-specific distribution. Band-pass filtering (0.008–0.09 Hz) will be applied within the same general linear model to avoid reintroducing noise. Datasets with mean FD > 0.2 mm or >20% censored volumes will be excluded. Global signal regression will not be used in the primary analysis given ongoing debate; identical models with GSR will be run as a sensitivity check.

Connectivity analyses will center on bilateral DLPFC seeds placed at the stimulation site (6-mm spheres in MNI space, inverse-warped to native to form subject-specific masks). *A priori* ROIs will further include sgACC, PCC/precuneus (DMN), anterior insula/dACC (salience), and intraparietal/inferior parietal nodes (frontoparietal/executive). For each seed, subject-level Pearson correlation maps will be Fisher-z transformed and averaged across runs; ROI-to-ROI z-transformed matrices will also be computed using the Schaefer parcellation and Yeo networks (Fox et al., 2005; Yeo et al., 2011; Schaefer et al., 2018). To improve specificity, all connectivity will be estimated after nuisance regression and scrubbing; where appropriate, surface-based analyses in fsaverage will additionally be performed. As secondary endpoints, we will summarize graph metrics (global/local efficiency, modularity, participation coefficient) from the ROI graphs.

Primary within-subject contrasts will evaluate pre- vs. post-stimulation changes in (i) DLPFC-sgACC coupling (*a priori* expectation: stronger anticorrelation post-stimulation), (ii) DLPFC interactions with executive and default-mode networks (hypothesized ECN-DMN rebalancing), and (iii) whole-brain seed-to-voxel effects from the stimulated DLPFC. Group inference will use mixed-effects GLMs; voxelwise maps will be thresholded with permutation testing (≥5,000 permutations, TFCE), and ROI-level tests will controll FDR *q* < 0.05. We will report effect sizes for paired contrasts (Cohen’s dz) and 95% CIs. Robustness will be assessed by repeating models with/without GSR and by varying DLPFC seed radius (4 - 8 mm).

### 2.13 Quality management

To ensure consistency and compliance throughout the study, a detailed manual of operations and procedures, along with a case report form will be developed based on the study protocol. These documents will standardize key processes, including participant recruitment, outcome measurement, data entry, and analysis. At the same time, comprehensive monitoring plans will be included to ensure participant safety and maintain data integrity.

Participants will receive detailed education about the study’s requirements to minimize protocol non-adherence. Investigators and research staff will undergo extensive training before study initiation, with continuous support and supervision provided during the study to ensure the accurate execution of all procedures.

Data management will prioritize security and confidentiality. Encrypted channels will be used for data transfer to prevent unauthorized access. All personal identifiers will be removed during data processing, ensuring complete anonymization and protecting participant privacy. All procedures will adhere to applicable ethical and legal standards for data management.

## 3 Discussion

Obesity is a multifaceted condition influenced by genetic predisposition and a range of environmental risk factors. Dysregulation of eating behavior is a significant contributor to the development of obesity. The DLPFC has been identified as a key region in regulating eating behavior and is integral to executive functions (Ester and Kullmann, 2022). Therefore, exploring brain-based strategies to rebalance the function of the DLPFC and enhance executive control over food intake may help accelerate the prevention and treatment of obesity, with potential positive impacts on the long-term management and prevention of obesity.

Low-intensity pulsed ultrasound is a novel, non-invasive brain stimulation and modulation technique that has emerged in recent years. Research on LIPUS modulation of brain neurons has expanded from rodents to non-human primates and humans. A small-sample clinical study on Alzheimer’s disease published in 2020 showed that pulsed ultrasound could modulate cortical and hippocampal neuron function in patients, improving cognitive memory and neuropsychological scores, with a safety profile indicating its efficacy as a neuromodulation tool (Beisteiner et al., 2020).

In this project, we propose a single-center, small-sample, randomized controlled double-blind clinical trial to evaluate the therapeutic potential of LIPUS on executive function in individuals with obesity over a 4-week treatment period. Meanwhile, our team has developed a wearable LIPUS device that enables patients to undergo treatment at home, eliminating the need for daily hospital visits and offering time-saving convenience. Additionally, to assess the effects of ultrasound modulation, we will use functional near-infrared brain imaging (fNIRS), electroencephalography (EEG), and functional magnetic resonance imaging (fMRI) to evaluate DLPFC excitability, microcirculation, and improvements in executive function among the obese population. By conducting this in-depth analysis, we aim to provide new perspectives and strategies for obesity management and inform future research directions.

We acknowledge certain limitations in our trial. First, it is a single-center trial with a relatively small sample size of individuals with obesity, which may limit the broader applicability of the findings. Additionally, the outcome measures are assessed only during or immediately after the LIPUS treatment, without a follow-up period to evaluate the long-term effects of the intervention. In future phases of the study, we plan to expand the study to a multi-center design and extend the follow-up period to assess the durability of treatment effects.

## References

[B1] Anderson E. C. Cantelon J. A. Holmes A. Giles G. E. Brunyé T. T. Kanarek R. (2023). Transcranial direct current stimulation (tDCS) to dorsolateral prefrontal cortex influences perceived pleasantness of food. *Heliyon* 9:e13275. 10.1016/j.heliyon.2023.e13275 36816290 PMC9929296

[B2] Beam W. Borckardt J. J. Reeves S. T. George M. (2009). An efficient and accurate new method for locating the F3 position for prefrontal TMS applications. *Brain Stimul.* 2 50–54. 10.1016/j.brs.2008.09.006 20539835 PMC2882797

[B3] Beisteiner R. Matt E. Fan C. Baldysiak H. Schönfeld M. Philippi Novak T. (2020). Transcranial pulse stimulation with ultrasound in Alzheimer’s disease: A new navigated focal brain therapy. *Adv. Sci.* 7:1902583. 10.1002/advs.201902583 32042569 PMC7001626

[B4] Bhattacharya A. Mrudula K. Sreepada S. S. Sathyaprabha T. Pal P. Chen R. (2022). An overview of noninvasive brain stimulation: Basic principles and clinical applications. *Can. J. Neurol. Sci.* 49 479–492. 10.1017/cjn.2021.158 34238393

[B100] Blechert J. Lender A. Polk S. Busch N. A. Ohla K. (2019). Food-pics_extended–an image database for experimental research on eating and appetite: Additional images, normative ratings and an updated review. *Front. Psychol.* 10:307. 10.3389/fpsyg.2019.00307 30899232 PMC6416180

[B5] Caballero B. (2019). Humans against obesity: Who will win? *Adv. Nutr.* 10 S4–S9. 10.1093/advances/nmy063 30721956 PMC6363526

[B6] Chan A. W. Tetzlaff J. M. Gøtzsche P. C. Altman D. G. Mann H. Berlin J. A. (2013). SPIRIT 2013 explanation and elaboration: Guidance for protocols of clinical trials. *BMJ* 346:e7586. 10.1136/bmj.e7586 23303884 PMC3541470

[B7] Dallapiazza R. F. Timbie K. F. Holmberg S. Gatesman J. Lopes M. Price R. (2018). Noninvasive neuromodulation and thalamic mapping with low-intensity focused ultrasound. *J. Neurosurg.* 128 875–884. 10.3171/2017.10.JNS171595 28430035 PMC7032074

[B8] Doebel S. (2020). Rethinking executive function and its development. *Perspect. Psychol. Sci.* 15 942–956. 10.1177/1745691620916787 32348707

[B9] Ester T. Kullmann S. (2022). Neurobiological regulation of eating behavior: Evidence based on non-invasive brain stimulation. *Rev. Endocrine Metab. Disord.* 23 753–772. 10.1007/s11154-021-09697-3 34862944 PMC9307556

[B10] Evans C. Bachmann C. Lee J. S. A. Gregoriou E. Ward N. Bestmann S. (2020). Dose-controlled tDCS reduces electric field intensity variability at a cortical target site. *Brain Stimul.* 13 125–136. 10.1016/j.brs.2019.10.004 31653475

[B11] Fabregat-Sanjuan A. Pàmies-Vilà R. Pascual-Rubio V. (2022). Evaluation of the Beam-F3 method for locating the F3 position from the 10-20 international system. *Brain Stimul.* 15 1011–1012. 10.1016/j.brs.2022.05.004 35863653

[B12] Foland-Ross L. C. Buckingam B. Mauras N. Arbelaez A. Tamborlane W. Tsalikian E. (2019). Executive task-based brain function in children with type 1 diabetes: An observational study. *PLoS Med.* 16:e1002979. 10.1371/journal.pmed.1002979 31815939 PMC6901178

[B13] Foldi C. J. Morris M. J. Oldfield B. J. (2021). Executive function in obesity and anorexia nervosa: Opposite ends of a spectrum of disordered feeding behaviour? *Prog. Neuropsychopharmacol. Biol. Psychiatry* 111:110395. 10.1016/j.pnpbp.2021.110395 34217755

[B14] Fomenko A. Chen K. S. Nankoo J. F. Saravanamuttu J. Wang Y. El-Baba M. (2020). Systematic examination of low-intensity ultrasound parameters on human motor cortex excitability and behavior. *eLife* 9:e54497. 10.7554/eLife.54497 33236981 PMC7728443

[B15] Fomenko A. Neudorfer C. Dallapiazza R. F. Kalia S. Lozano A. (2018). Low-intensity ultrasound neuromodulation: An overview of mechanisms and emerging human applications. *Brain Stimul.* 11 1209–1217. 10.1016/j.brs.2018.09.001 30166265

[B16] Fox M. D. Snyder A. Z. Vincent J. L. Corbetta M. Van Essen D. C. Raichle M. E. (2005). The human brain is intrinsically organized into dynamic, anticorrelated functional networks. *Proc. Natl. Acad. Sci. U S A.* 102 9673–9678. 10.1073/pnas.0504136102 15976020 PMC1157105

[B17] Ghobadi-Azbari P. Malmir N. Vartanian M. Mahdavifar-Khayati R. Robatmili S. Hadian V. (2022). Transcranial direct current stimulation to modulate brain reactivity to food cues in overweight and obese adults: study protocol for a randomized controlled trial with fMRI (NeuroStim-Obesity). *Trials* 23:297. 10.1186/s13063-022-06234-8 35413923 PMC9003175

[B18] Gluck M. E. Viswanath P. Stinson E. J. (2017). Obesity, appetite, and the prefrontal cortex. *Curr. Obes. Rep.* 6 380–388. 10.1007/s13679-017-0286-3 29071480

[B19] Hayner M. Hynynen K. (2001). Numerical analysis of ultrasonic transmission and absorption of oblique plane waves through the human skull. *J. Acoust. Soc. Am.* 110 3319–3330. 10.1121/1.1410964 11785832

[B20] He Q. Huang X. Zhang S. Turel O. Ma L. Bechara A. (2019). Dynamic causal modeling of insular, striatal, and prefrontal cortex activities during a food-specific Go/NoGo task. *Biol. Psychiatry Cogn. Neurosci. Neuroimaging* 4 1080–1089. 10.1016/j.bpsc.2019.07.003 30691967 PMC6609512

[B21] Jornada M. N. D. Antunes L. C. Alves C. Torres I. L. S. Fregni F. Sanches P. R. (2024). Impact of multiple-session home-based transcranial direct current stimulation (M-HB-tDCS) on eating behavior in fibromyalgia: A factorial randomized clinical trial. *Brain Stimul.* 17 152–162. 10.1016/j.brs.2024.02.001 38336340

[B22] Kalanthroff E. Davelaar E. J. Henik A. Goldfarb L. Usher M. (2018). Task conflict and proactive control: A computational theory of the Stroop task. *Psychol. Rev.* 125 59–82. 10.1037/rev0000085 29035077

[B23] Kang H. (2021). Sample size determination and power analysis using the G*Power software. *J. Educ. Eval. Health Prof.* 18:17. 10.3352/jeehp.2021.18.17 34325496 PMC8441096

[B24] Kekic M. McClelland J. Campbell I. Nestler S. Rubia K. David A. (2014). The effects of prefrontal cortex tDCS on food craving and temporal discounting in women with frequent food cravings. *Appetite* 78 55–62. 10.1016/j.appet.2014.03.017 24656950

[B25] Kim S. H. Chung J. H. Kim T. H. Lim S. Kim Y. Lee Y. (2018). The effects of rTMS on eating behaviors and body weight in obesity: A randomized controlled study. *Brain Stimul.* 11 528–535. 10.1016/j.brs.2017.12.008 29326022

[B26] Kringelbach M. L. de Araujo I. E. Rolls E. T. (2004). Taste-related activity in the human dorsolateral prefrontal cortex. *Neuroimage* 21 781–788. 10.1016/j.neuroimage.2003.09.016 14980581

[B27] Labonté K. Nielsen D. E. (2023). Measuring food-related inhibition with go/no-go tasks: Critical considerations for experimental design. *Appetite* 185:106497. 10.1016/j.appet.2023.106497 36893916

[B28] Le D. S. Pannacciulli N. Chen K. Salbe A. Del Parigi A. Hill J. (2007). Less activation in the left dorsolateral prefrontal cortex in obese than in lean women and its association with successful weight loss. *Am. J. Clin. Nutr.* 86 573–579. 10.1093/ajcn/86.3.573 17823419 PMC2128057

[B29] Legon W. Adams S. Bansal P. Patel P. Hobbs L. Ai L. (2020). A retrospective qualitative report of symptoms and safety from transcranial focused ultrasound for neuromodulation in humans. *Sci. Rep.* 10:5573. 10.1038/s41598-020-62265-8 32221350 PMC7101402

[B30] Lin X. Li H. (2021). Obesity: Epidemiology, pathophysiology, and therapeutics. *Front. Endocrinol.* 12:706978. 10.3389/fendo.2021.706978 34552557 PMC8450866

[B31] Lowe C. J. Reichelt A. C. Hall P. A. (2019). The prefrontal cortex and obesity: A health neuroscience perspective. *Trends Cogn. Sci.* 23 349–361. 10.1016/j.tics.2019.02.007 30824229

[B32] Mattson M. P. (2019). An evolutionary perspective on why food overconsumption impairs cognition. *Trends Cogn. Sci.* 23 200–212. 10.1016/j.tics.2019.01.002 30670325 PMC6412136

[B33] Obesity G. Collaborators. (2024). Worldwide trends in underweight and obesity from 1990 to 2022: A pooled analysis of 3663 population-representative studies with 222 million children, adolescents, and adults. *Lancet* 403 1027–1050. 10.1016/S0140-6736(23)02094-3 38432237 PMC7615769

[B34] Obrig H. Villringer A. (2003). Beyond the visible—imaging the human brain with light. *J. Cereb. Blood Flow Metab.* 23 1–18. 10.1097/01.WCB.0000043472.45775.29 12500086

[B35] Pellow C. Pichardo S. Pike G. B. (2024). A systematic review of preclinical and clinical transcranial ultrasound neuromodulation and opportunities for functional connectomics. *Brain Stimul.* 17 734–751. 10.1016/j.brs.2024.06.005 38880207

[B36] Piché M. E. Tchernof A. Després J. P. (2020). Obesity phenotypes, diabetes, and cardiovascular diseases. *Circ. Res.* 126 1477–1500. 10.1161/CIRCRESAHA.120.316242 32437302

[B37] Qin H. Luo Z. Sun Y. He Z. Qio B. Chen Y. (2023). Low-intensity pulsed ultrasound promotes skeletal muscle regeneration via modulating the inflammatory immune microenvironment. *Int. J. Biol. Sci.* 19 1123–1145. 10.7150/ijbs.78225 36923940 PMC10008697

[B38] Raud L. Westerhausen R. Dooley N. Huster R. (2020). Differences in unity: The go/no-go and stop signal tasks rely on different mechanisms. *Neuroimage* 210:116582. 10.1016/j.neuroimage.2020.116582 31987997

[B39] Rösch S. A. Schmidt R. Lührs M. Ehlis A. Hesse S. Hilbert A. (2020). Evidence of fNIRS-based prefrontal cortex hypoactivity in obesity and binge-eating disorder. *Brain Sci.* 11:19. 10.3390/brainsci11010012 33375315 PMC7823505

[B40] Schaefer A. Kong R. Gordon E. M. Laumann T. O. Zuo X. N. Holmes A. J. (2018). Local-global parcellation of the human cerebral cortex from intrinsic functional connectivity MRI. *Cereb. Cortex* 28 3095–3114. 10.1093/cercor/bhx179 28981612 PMC6095216

[B41] Schlögl H. Horstmann A. Villringer A. Stumvoll M. (2016). Functional neuroimaging in obesity and the potential for development of novel treatments. *Lancet Diabetes Endocrinol.* 4 695–705. 10.1016/S2213-8587(16)30036-0 26838265

[B42] Seravalle G. Grassi G. (2017). Obesity and hypertension. *Pharmacol. Res.* 122 1–7. 10.1016/j.phrs.2017.05.017 28532816

[B43] Shimokawa H. Shindo T. Ishiki A. Tomita N. Ichijyo S. Watanabe T. (2022). A pilot study of whole-brain LIPUS therapy for early stage of Alzheimer’s disease. *Tohoku J. Exp. Med.* 258 167–175. 10.1620/tjem.258.167 36104179

[B44] Stinson E. J. Travis K. T. Magerowski G. Alonso-Alonso M. Krakoff J. Gluck M. (2022). Improved food Go/No-Go scores after prefrontal tDCS in a randomized trial. *Obesity* 30 2005–2013. 10.1002/oby.23529 36052819

[B45] Su W. S. Wu C. H. Chen S. F. Yang F. (2017). Low-intensity pulsed ultrasound improves behavioral and histological outcomes after traumatic brain injury. *Sci. Rep.* 7:15524. 10.1038/s41598-017-15774-7 29138458 PMC5686128

[B46] Tufail Y. Yoshihiro A. Pati S. Li M. Tyler W. (2011). Ultrasonic neuromodulation by brain stimulation with transcranial ultrasound. *Nat. Protoc.* 6 1453–1470. 10.1038/nprot.2011.371 21886108

[B47] U.S. Food and Drug Administration. (2019). *Guidance for industry and FDA staff: information for manufacturers seeking marketing clearance of diagnostic ultrasound systems and transducers.* Silver Spring, MD: U.S. Department of Health and Human Services.

[B48] Ulrich M. Steigleder L. Grön G. (2016). Neural signature of the food craving questionnaire (FCQ)-Trait. *Appetite* 107 303–310. 10.1016/j.appet.2016.08.045 27524657

[B49] Vergallito A. Feroldi S. Pisoni A. Romero Lauro L. J. (2022). Inter-individual variability in tDCS effects: A narrative review on the contribution of stable, variable, and contextual factors. *Brain Sci.* 12:522. 10.3390/brainsci12050522 35624908 PMC9139102

[B50] Wang K. Xu L. Huang T. Meng F. Yang Q. Deng Z. (2024). Food-related inhibitory control deficits in obesity: ERP evidence from a go/no-go task. *Physiol. Behav.* 281:114573. 10.1016/j.physbeh.2024.114573 38685523

[B51] Wang W. Li Z. Yan Y. Wu S. Yao X. Gao C. (2024). LIPUS-induced neurogenesis: A potential therapeutic strategy for TBI. *Exp. Neurol.* 371:114588. 10.1016/j.expneurol.2024.114588 37907126

[B52] Welch P. D. (1967). The use of fast fourier transform for the estimation of power spectra. *IEEE Trans. Audio Electroacoust.* 15 70–73. 10.1109/TAU.1967.1161901

[B53] Wen H. Yang C. Shang T. Pang Y. (2024). Electrophysiological and behavioral differences of general and food-specific inhibitory control in intuitive eating. *Appetite* 199:107402. 10.1016/j.appet.2023.107402 38754767

[B54] Yang Y. Shields G. S. Guo C. Liu Y. (2018). Executive function performance in obesity and overweight individuals: A meta-analysis. *Neurosci. Biobehav. Rev.* 84 225–244. 10.1016/j.neubiorev.2017.11.022 29203421

[B55] Yeo B. T. T. Krienen F. M. Sepulcre J. Sabuncu M. R. Lashkari D. Hollinshead M. (2011). The organization of the human cerebral cortex estimated by intrinsic functional connectivity. *J. Neurophysiol*. 106, 1125–1165. 10.1152/jn.00338.2011 21653723 PMC3174820

[B56] Zeng K. Darmani G. Fomenko A. Xia X. Tran S. Nankoo J. (2022). Induction of human motor cortex plasticity by theta burst transcranial ultrasound stimulation. *Ann. Neurol.* 91 238–252. 10.1002/ana.26085 34964172

[B57] Zeng Q. Li N. Pan X. F. Chen L. Pan A. (2021). Clinical management and treatment of obesity in China. *Lancet Diabetes Endocrinol.* 9 393–405. 10.1016/S2213-8587(21)00092-0 34022157

[B58] Zhang T. Pan N. Wang Y. Liu C. Hu S. (2021). Transcranial focused ultrasound neuromodulation: A review of the excitatory and inhibitory effects on brain activity in human and animals. *Front. Hum. Neurosci.* 15:749162. 10.3389/fnhum.2021.749162 34650419 PMC8507972

